# The Lunatic at Large

**Published:** 1899-09-30

**Authors:** 


### The Lunatic at Large.

It is rarely that a week passes without some fresh example being brought to our notice illustrating the vagaries of the lunatic at large, and showing the danger to which not only he but the community are exposed by the freedom from control which he too often enjoys. A week or two ago a medical practitioner was furiously attacked by a man who turned out to be liable to more or less transient paroxysms of epileptic mania, and, although in this particular instance no special harm was done, it was obvious from the circumstances of the case that it was by mere chance that the outburst did not take a far more serious and more criminal form.

Last Monday a pensioner, who had been strange in his manner for some time past, made a murderous attack upon an old gardener at Warwick. The old man had been working in his garden allotment, which adjoined that of his assailant, when the latter attacked him suddenly with a garden fork, piercing his skull. The madman escaped to his home, where he cut his throat. Now, this is the sort of thing which is happening continually, and it becomes a matter of very serious public concern that all that is possible should be done to diminish the number of wandering lunatics.

We need hardly say that the whole subject is surrounded with many difficulties, first among which is that which arises from lack of accommodation in consequence of the increasing number of lunatics. Many of them were idiots and helpless imbeciles, whose friends are anxious to hand them over to the care of the asylum authorities. Another difficulty arises from the dislike which many medical men have to "certifying" in the early stages of insanity, the consequence of their timidity in this respect being that in many cases, before an insane person can be restrained, he must for a period, and sometimes for no inconsiderable period, have been a lunatic at large. Finally, we have the not inconsiderable number of insane persons whose friends hate the thought of an asylum, and will do anything rather than have them placed in an institution, or even have them "certified," so great is their dislike to branding the patient—but often still more to branding the family—with the stain of insanity. As Dr. Savage says, "we may preach as we like and advise as we will, yet among the wealthy classes in particular, and among those with numerous relations—brothers and sisters particularly—we cannot get consent to early removal to asylum care. The friends object to the word 'asylum,' and they have got to have almost as strong a prejudice against a relation becoming a 'certified lunatic.'" Now this is a great difficulty and an urgent danger. Patients who ought to be safely taken care of in asylums are being treated without certificate in houses in the country without proper medical supervision, and with but little security against the violence of their attendants. Patients are being hidden away by unscrupulous persons who, for the sake of the heavy fees offered, will run the risk of prosecution by the Lunacy Commissioners, and it is impossible to say what is not being done to them; and, lastly, patients insane in a less degree are being sent off travelling in the hope that "distraction" and "changes of scene" may restore their unstable minds, and such patients, until they commit suicide, which they too often do, are "lunatics" very much "at large" indeed. To get over this difficulty a Bill was drawn up during the past session to enable a medical man to place a patient in private care, for a period not exceeding six months, if he could certify that he was suffering from mental disease which was not of a confirmed character. Unfortunately, this Bill was set aside But if such a Bill could be passed, it would do much to enable many patients to be brought under control at an earlier stage of their malady than is the case at present. The plan works well in Scotland, and with the ample safeguards with which it is surrounded in the Bill proposed it ought to be useful in England also. Within a day of signing such a certificate a medical man must send a copy of it to the Commissioners, who may visit the patient. The person in charge must also give notice of receiving the patient, of any change of address, of the discharge or death of the patient, and, finally, a patient dealt with in this manner cannot be certified again within two years of the expiration of the certificate. The suggested legislation would only touch one side of a wide problem, but it would do something to diminish the number of lunatics at large.

